# Comparison of Atherosclerotic Plaque Compositions in Diabetic and Non-diabetic Patients

**DOI:** 10.7759/cureus.45721

**Published:** 2023-09-21

**Authors:** Mustafa Ozan Çakır, Mustafa Taner Gören

**Affiliations:** 1 Department of Cardiology, Bulent Ecevit University Faculty of Medicine, Zonguldak, TUR; 2 Department of Cardiology, Istanbul University School of Medicine, Istanbul, TUR

**Keywords:** hemoglobin a1c protein, atherosclerosis, atherosclerotic plaque, coronary artery disease, diabetes mellitus

## Abstract

Introduction: Diabetes mellitus is one of the major risk factors for coronary artery disease. Intravascular ultrasound (IVUS) imaging has an important role in the evaluation of atherosclerotic coronary artery disease. The aim of the study was to investigate the potential link between diabetes mellitus and plaque vulnerability in patients with coronary artery disease.

Methods: In total, 26 patients with acute coronary syndrome (eight with diabetes mellitus) and 34 with stable angina pectoris (16 with diabetes mellitus) constituted the study population. Patients underwent IVUS ultrasound and virtual histology (VH)-IVUS imaging during routine diagnostic catheterization procedures. A total of 70 plaques in 60 patients were examined.

Results: Patients with diabetes mellitus had a significantly greater percentage of fibrofatty components in the minimal lumen area (MLA) (17 ± 12 in diabetics; 12 ± 6 in non-diabetics; p=0.06). Thin-cap fibroatheromas were more frequent in patients with diabetes mellitus (72% versus 45%; p=0.012). There was a positive correlation between the presence of attenuated plaque and hemoglobin A1C (HbA1c) levels as well (7.09 ± 1.66 versus 6.02 ± 1.00; p=0.011). Patients with HbA1C ≥7.5% also had the highest prevalence of attenuated plaque.

Conclusion: As shown by VH-IVUS, the prevalence of vulnerable plaques in patients with diabetes mellitus was much higher than that in non-diabetic patients. The presence of attenuated plaque detected in grayscale intravascular ultrasonography was associated with high HbA1C levels in diabetic patients. Diabetes mellitus may cause cardiovascular vulnerability by changing the plaque morphology.

## Introduction

Diabetes mellitus (DM) is considered to be the equivalent of coronary heart disease because people with DM have the same lifetime risk of cardiovascular events as non-diabetic people who have had myocardial infarction [1]. About 75% of patients with type 2 diabetes die from cardiovascular disease [2]. In addition, patients with type 2 diabetes are exposed to more silent myocardial ischemia and worse outcomes after acute coronary syndromes than non-diabetic patients [3]. The mechanisms of cardiovascular disease (a macrovascular complication of DM) and why such poor outcomes occur in patients without diabetes have not been fully elucidated.

Despite major advances in the diagnosis and treatment of coronary heart disease, sudden deaths mostly occur in patients with no previous symptoms [4]. In a retrospective autopsy series, thrombotic coronary death and acute coronary syndrome were found to be related to the characteristics of the plaque and related factors [5]. Coronary plaques that are prone to cracking or erosion and have a high potential to cause acute coronary syndrome and sudden death are defined as vulnerable plaques. The common characteristics of these plaques in postmortem evaluation are a thin fibrous capsule (<65μm), a large pool of lipids, and increased macrophage activity [6].

Although coronary angiography is used as a primary method for determining the anatomical severity of coronary artery disease, its accuracy has been questioned in many studies [7,8]. Clinically significant intra- and inter-observer variability in the interpretation of angiography was revealed [9]. Intravascular ultrasound (IVUS) imaging with a tomographic viewing angle has a great advantage in widely diseased segments, bifurcation points, ostial regions, and eccentric locations where it is difficult to evaluate the severity of atherosclerosis with conventional angiography [10]. With IVUS examination, measurements related to the lumen, outer elastic membrane, atheroma plaque, and virtual histological measurements can be made. Intravascular ultrasound is accepted as the gold standard modality for examining the coronary artery vessel wall in vivo [11].

In this study, we wanted to compare the composition of coronary artery lesions determined by IVUS according to their morphological and virtual-histological features and their differences in terms of vulnerability in diabetic and non-diabetic patients.

## Materials and methods

Study population

In this study, 34 patients with a diagnosis of stable angina pectoris who applied to the outpatient clinic of the Istanbul University Faculty of Medicine, Department of Cardiology, Istanbul, Turkey, between June 2012 and June 2013, who had an indication for elective coronary angiography and those who had 50% or 70% stenosis in at least one coronary artery in coronary angiography, and 26 patients who applied to our clinic due to acute coronary syndrome and underwent coronary angiography and intravascular ultrasonography were included. A total of 70 coronary lesions in 60 patients were examined. All patients were given acetylsalicylic acid at admission. During the procedure for patients requiring percutaneous coronary intervention, a loading dose of 600 mg clopidogrel was given at the time of diagnosis to patients diagnosed with acute coronary syndrome, and low molecular weight heparin was added to the treatment of all patients during their hospitalization after the procedure. Ethical approval was obtained from the Istanbul University Faculty of Medicine Clinical Research Ethics Committee (approval number: 2012-650-06/06). Informed consent was obtained from all patients.

Intravascular ultrasound examination

The IVUS examination was applied to all patients. Before percutaneous coronary intervention, after completion of hemodynamic measurements, while the guide wire in the responsible vessel was distal to the stenosis, a 2.9 Fr, 20 MHz IVUS catheter (Eagle Eye Gold, Volcano Corp., Rancho Cordova, CA, USA) was advanced approximately 10 mm distal to the stenosis. The catheter connected to the IVUS console (IVUS3 system, Volcano Therapeutics, Philips Volcano, San Diego, CA, USA) was withdrawn with an automatic motorized retraction device at a speed of 0.5 mm/s, and gray-scale IVUS and virtual histology (VH)-IVUS images were recorded along the relevant segment. After reaching a sufficient number of patients, the images stored in the memory of the IVUS console were examined. The narrowest lumen area (MLA) was selected as the cross-section, and the calculation of the external elastic membrane (EEM) cross-sectional area (CA), the MLA, the plate + media layer cross-sectional area (P+M CA), and plaque load were planned on this cross-sectional area. Examined sections were classified into four different groups according to their histopathological characters with VH-IVUS technology, and the absolute values in mm² and proportional percentage values were calculated separately for each of the groups separated by color coding. The patient segment was then determined in the relevant vessel, along with the lesion length and total plaque volume, and the absolute and percentage values of individual plaque types according to tissue characteristics throughout the lesion and evaluation in terms of vulnerability criteria.

Statistical analysis

Statistical analyses were performed using the IBM SPSS software (IBM Corp., Armonk, NY, USA) program. The classified data were compared numerically or by expressing their frequency using the chi-square test. Measurements were compared at each step with a paired t-test. Group means were compared with the Student's t-test for independent groups. Relationships between variables were evaluated using Pearson's correlation analysis or linear regression analysis. Establishing a multivariate logistic regression model to detect independent predictors of hemoglobin A1C (HbA1C) change, parameters included as variables were age, diabetes mellitus, hypertension, and low-density lipoprotein (LDL) cholesterol level. A p-value of < 0.05 was considered significant.

## Results

The mean age of the 60 patients (44 males and 16 females) included in the study was 58.9 ± 10.0 years. A total of 70 lesions in these 60 patients were examined. In terms of clinical features, there were 25 diabetic and 35 non-diabetic patients. A total of 30 lesions belonged to the patients followed up with the diagnosis of diabetes mellitus (42%), 54 lesions with the diagnosis of hypertension (77%), and 36 lesions with the diagnosis of dyslipidemia (51%). Thirty-five (50%) of the lesions were present in patients who were smokers. Twenty-six lesions were examined in 26 patients who were admitted to the coronary angiography laboratory with the diagnosis of non-ST elevation myocardial infarction, and 44 lesions were examined in 34 patients who were diagnosed with stable angina pectoris and were admitted to the coronary angiography laboratory.

When the patient population was analyzed, diabetic patients were older than non-diabetic patients (57.3 vs. 60.8 years, p=0.034). When both patient groups were compared in terms of body mass indices, it was seen that diabetic patients were more obese. (The mean body mass index was 30.8 kg/m^2^ in diabetics and 26.8 kg/m^2^ in non-diabetic patients, with a p-value of 0.002). In non-diabetic patients, the history of previous myocardial infarction was not significantly higher (27% vs. 13% patients; p= 0.153). Only patients with a history of previous myocardial infarction were receiving long-term statin therapy. Statin treatment was initiated in patients admitted to our hospital with a diagnosis of acute coronary syndrome in accordance with the relevant guideline recommendations. There was no statistically significant difference between long-term statin use rates (p= 0.153).

Angiographic features

Thirty-six lesions were in the left anterior descending artery (51%), 12 lesions were in the circumflex artery (17%), 17 lesions were in the right coronary artery (24%), one lesion was in the diagonal artery (1%), and four lesions were in the intermediate artery (5%). Single-vessel disease was present in 66% of the patients. Successful bare metal stent implantation was performed on the responsible lesion in all patients presenting with acute coronary syndrome. Predilatation was performed before stent implantation in 24 patients (63%), and direct stent implantation was performed in 14 (37%) patients. The mean stent length in patients with acute coronary syndrome was 19.3 ± 5.3 mm.

Between the diabetic and non-diabetic groups, there was no significant difference in the number of lesions requiring percutaneous coronary intervention, mean stent length, or mean stent diameter (Table 1).

**Table 1:** Demographic and angiographic characteristics n: number of lesions, HbA1C: hemoglobin A1C; LDL: low-density lipoproteins; HDL: high-density lipoprotein; LAD: left anterior descending artery; RCA: right coronary artery; Cx: circumflex artery; PCI: percutaneous coronary intervention

Demographic and angiographic characteristics (n = 70 lesions)	Diabetic lesions (n=30)	Non-diabetic lesions (n=40)	p-value
Age, mean (year)	60.8 ± 6.8	57.3 ± 12.4	0.034
Male/all patients, n, (%)	19/30 (63%)	32/40 (80%)	0.059
Body mass index, average	30.8 ± 4.1	26.8 ± 3.8	0.002
Acute coronary syndrome, n, (%)	8/26 (30%)	18/26 (70%)	0.014
Previous myocardial infarction, n (%)	4 (13%)	11(%27)	0.153
Hypertension, n %	26/30 (86%)	28/40 (70%)	0.100
Chronic kidney disease, n, (%)	1/30 (3%)	8/40 (20%)	0.072
HbA1C, (%)	7.98 ± 1.5	5.74 ± 0.4	0.001
Total cholesterol, mean(mg/dl)	186 .± 48.7	203.2 ± 48.1	0.254
Triglyceride, mean (mg/dl)	166.0 ± 118.4	160.4 ± 72.7	0.918
LDL, mean (mg/dl)	113.5 ± 35.8	130.3 ± 39.4	0.166
HDL, mean (mg/dl)	38.2 ± 8.6	41.4 ± 9.8	0.354
Smoking habit, n, (%)	10/30 (33%)	25/40 (62%)	0.129
Insulin usage, n, (%)	14 (46%)	0	-
Oral antidiabetic therapy, n, (%)	24 (80%)	0	-
Ejection fraction, mean (%)	60.3 ± 7	59.1 ± 7.7	0.919
LAD Stenting, n, (%)	16 (53%)	20 (50%)	-
Diagonal artery stenting, n, (%)	1 (3%)	0 (0%)	-
Intermediary artery stenting, n, (%)	0 (0%)	4 (10%)	-
Cx stenting, n, (%)	3 (10%)	9 (22%)	-
RCA stenting, n, (%)	10 (33%)	7 (17%)	-
PCI performed lesion, n, (%)	20/30 (66%)	29/40 (72%)	0.904
Stent diameter, mean (mm)	2.9	3.1	0.281
Stent length, mean (mm)	21	21	1.000

Relationship of plaque morphology and diabetes mellitus

A total of 70 lesions (30 diabetic and 40 non-diabetic) included in the study were evaluated with a grayscale and IVUS. The angiographic and demographic characteristics of the patients are summarized in Table 1.

Most of the responsible lesions were in the left anterior descending artery in both the diabetic (53%) and non-diabetic (50%) groups. Stent implantation was performed in 20 lesions (66%) in the diabetic group and 29 lesions (72%) in the non-diabetic group. There was no difference in stent diameters (2.9 mm in diabetic lesions versus 3.1 mm in non-diabetic lesions) and lengths (21 mm in diabetic lesions versus 21 mm in non-diabetic lesions) applied in both groups (p > 0.05).

Virtual histology-intravascular ultrasound examinations, grayscale, and VH-IVUS examinations were performed by examining the images stored in the memory of the IVUS console after recording during the procedure.

In the non-diabetic group, the mean EEM CA was 13.0 ± 4.8 mm², the plaque load was 77.8 ± 7.9 mm^2^, and the MLA was 2.6 ± 0.9 mm². The mean lesion length was 14.5 ± 7.4 mm, and the total plaque volume was 129.0 ± 79.2 mm³. As a result of VH-IVUS examinations in the lowest lumen area, mean fibrous CA was 4.6 ± 2.6 mm² (57.0% ± 14.0%), fibrous lipid CA was 1.0 ± 1.1 mm² (12.4% ± 8.0%), necrotic core CA was 1.6 ± 1.0 mm² (21.0% ± 7.9%), and dense calcium CA was 0.5 ± 0.5 mm² (9.0% + 11.9%). The necrotic core-dense calcium CA ratio was calculated as 5.0 ± 4.2. In segmental VH-IVUS analysis, mean fibrous volume was 50.7 ± 36.0 mm³ (55.9% ± 10.8%), fibrous fatty volume was 11.0 ± 10.7 mm³ (12.0% ± 6.5%), necrotic core volume (NCV) was 20.8 ± 16.9 mm³ (22.0% ± 6.4%), and dense calcium volume was 7.7 ± 9.6 (9.7% ± 10.2%). There was no significant difference in remodeling indices between the diabetic and non-diabetic plaque groups (1.06 ± 0.28 in the diabetic group; 1.14 ± 0.34 in the non-diabetic group; p=0.327) (Table 2).

**Table 2:** Intravascular ultrasound characteristics EEM CA: external elastic membrane cross-sectional area; CA: cross-sectional area; MLA: minimal lumen area; NC/DC: necrotic core/dense core Values are expressed as mean ± standard deviation

Gray scale measurements	Unit	Diabetic lesions	Non-diabetic lesions	p-value
EEM CA	(mm^2^)	12.2 ± 3.5	13.0 ± 4.8	0.2
Remodeling index	(mm^2^)	1.1 ± 0.3	1.0 ± 0.3	0.3
Plaque burden	(%)	78.7 ± 5.4	77.8 ± 7.9	0.7
MLA	(mm^2^)	2.4 ± 0.5	2.6 ± 0.9	0.2
Average lesion length	(mm)	15.4 ± 9.7	14.5 ± 7.4	0.6
Total plaque volume	(mm^3^)	127.6 ± 89.5	129.0 ± 79.2	0.4
Virtual histology measurements, measurements for the minimal lumen area	Unit	Diabetic lesions	Non-diabetic lesions	p-value
Fibrous CA	(mm^2^)	3.7 ± 1.6	4.6 ± 2.6	0.05
	(%)	52.9 ± 14.2	57.0 ± 14.0	0.9
Fibrous-fatty CA	(mm^2^)	1.1 ± 1.1	1.0 ± 1.1	0.6
	(%)	15.0 ± 15.2	12.4 ± 8.0	0.01
Necrotic core CA	(mm^2^)	1.6 ± 1.1	1.6 ± 1.0	0.8
	(%)	21.5 ± 10.5	21.0 ± 7.9	0.8
Dense calsium CA	(mm^2^)	0.6 ± 0.6	0.5 ± 0.5	0.3
	(%)	8.8 ± 8.4	9.0 ± 11.9	0.3
NC/DC		4.5 ± 4.4	5.0 ± 4.2	0.7
Segmental virtual histology analysis	Unit	Diabetic lesions	Non-diabetic lesions	p-value
Fibrous volume	(mm^3^)	45.4 ± 34.8	50.7 ± 36.0	0.8
	(%)	52.2 ± 10.3	55.9 ± 10.8	0.9
Fibrofatty volume	(mm^3^)	12.3 ± 10.8	11.0 ± 10.7	0.7
	(%)	16.6 ± 14.5	12.0 ± 6.5	0.06
Necrotic core volume	(mm^3^)	19.7 ± 19.4	20.8 ± 16.9	0.98
	(%)	20.6 ± 9.1	22.0 ± 6.4	0.5
Dense calcium volume	(mm^3^)	9.7 ± 11.2	7.7 ± 9.6	0.13
	(%)	9.8 ± 7.5	9.7 ± 10.2	0.44
NC/DC		3.4 ± 2.7	4.3 ± 3.2	0.3

The volume percentages of fibrous fatty plaque present in the segmental area in diabetic and non-diabetic patients were compared. In the diabetic group, there was a tendency to increase this component of the plaque, but no statistically significant difference was found (16.6% ± 14.5% in diabetics, 12.0% ± 6.5% in the non-diabetic group; p=0.060). The percentage of fibrous fatty tissue in the MLA measured by VH-IVUS was significantly higher in diabetic patients than in non-diabetic patients (15,0% ± 15,2% in diabetics, 12-4% ± 8% in non-diabetics; p= 0.013).

Both groups were evaluated in terms of thin fibrous encapsulated fibroatheroma (TCFA). Thin fibrous encapsulated fibroatheroma was found at a rate of 72% in the diabetic population and 45% in the non-diabetic group. The frequency of TCFA in diabetic patients was significantly higher (p=0.012) (Table 3).

**Table 3:** Plaque morphology of the patients

Plaque morphology (n=70 lesions)	Diabetic plaque, n=30 lesions (%)	Non-diabetic plaque, n=40 lesions (%)	p-value
Thick-cap fibrous fibroatheroma	1/30 (3%)	3/40 (7%)	0,901
Thin-cap fibrous fibroatheroma	22/30 (72%)	18/40 (45%)	0.012
Calcific thin fibrous capsulated fibroatheroma	11/30 (35%)	9/40 (22%)	0.205
Fibrocalsific plaque	4/30 (13%)	11/40 (27%)	0.154
Attenuated plaque	13/30 (43%)	10/40 (25%)	0.050
Attenuated plaque angle	13.7 + 26.7	16.5 + 25.0	0.603

Plaques seen in non-diabetic patients tended to be more fibrocalcific than plaques found in diabetic patients (27% vs. 13%). However, this finding was not statistically significant (p= 0.154). The two patient groups were evaluated for the presence of calcific TCFA, and it was seen that calcific TCFA was higher in diabetics (35% versus 22%). However, this finding was not statistically significant (p=0.205).

While the rate of attenuated plaque detected in grayscale IVUS examinations in the diabetic patient group was 43%, this rate was 25% in non-diabetic patients. The attenuated plaque rate tended to be higher in diabetic patients, but there was no statistically significant difference between the two groups (p=0.050).

The lesions of diabetic patients were divided into two groups: insulin users and non-insulin users. We detected fibroatheromas with thin fibrous caps in 12 (85%) of 14 lesions in patients who were not under the influence of insulin and in four (40%) of 10 lesions in patients under the influence of insulin. The statistical difference between the two groups was significant (p=0.031).

The relationship between IVUS findings and HbA1C levels

A total of 18 patients with attenuated plaques had higher HbA1C levels (7.09% + 1.66% vs. 6.02% + 1.00%). This difference was statistically significant between both groups (p=0.011).

**Figure 1:**
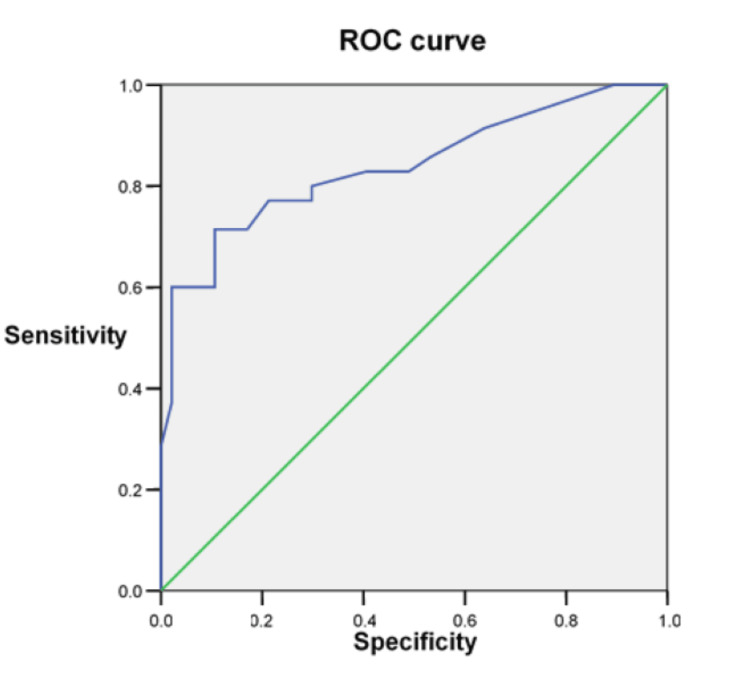
HbA1C value and attenuated plaque-ROC curve Based on the HbA1C level of 7.5, the area under the curve was found to be 0.83 in the evaluation made with the ROC curve. HbA1C: hemoglobin A1C; ROC curve: receiver operating characteristic curve

Based on the HbA1C level of 7.5%, the area under the curve (AUC) was found to be 0.83 in the evaluation made with the receiver operating characteristic (ROC) curve. As a result of the statistical evaluation, the detection of HbA1C >7.5% indicated the presence of attenuated plaque with 65% sensitivity and 90% specificity (Figure 1).

The mean HbA1C level was 6.95% + 1.61% in a patient with thin fibrous capsular fibroatheroma, while the mean HbA1C level was 6.47% + 1.42% in a patient without TCFA. There was no significant difference between HbA1C levels and the presence of thinly encapsulated fibroatheroma (p=0.240).

In the virtual intravascular ultrasonographic evaluation, the necrotic core/dense calcium (NC/DC) volume ratio (NCV/DCV) obtained by dividing the necrotic core volume by the dense calcium volume was found to be statistically related to the HbA1C level (r:0.336, p:0.005).

## Discussion

In our study, in which diabetic and non-diabetic coronary plaques were compared in terms of basic vulnerability criteria, the incidence of TCFA, which we know is an important intravascular ultrasonographic vulnerability criterion, was found to be significantly higher in the diabetic patient group compared to non-diabetic patients. In the diabetic patients who do not use insulin compared to the diabetic patients using insulin, a significant increase was found in the frequency of TCFA. The percentage of fibrous fatty tissue area (FFA%) measured in the MLA determined by IVUS was significantly higher in diabetic patients than in non-diabetic patients. In addition, the high HbA1C levels of the patients were related to the presence of attenuated plaque and the NC/DC ratio obtained by dividing the volume of the necrotic core by the dense calcium volume detected by using IVUS.

Diabetes mellitus has long been known to be an independent risk factor for atherosclerosis. However, in recent years, it has been understood that the clinical pictures called diabetes and metabolic syndrome, which is considered its precursor today, insulin resistance, and impaired fasting glucose are more serious risk factors than previously thought. A diabetic atherosclerotic lesion has an active inflammation process in which the number and activity of macrophages and lymphocytes increase in the atheroma plaque [12]. As a result of the interactions of leukocytes, vascular smooth muscle cells, platelets, and endothelial cells, tissue factor (TF) and various cytokines increase in plaque. Smooth muscle cell apoptosis is stimulated, and their number in plaque decreases, and collagen production in these cells also decreases [13,14]. Increased matrix metalloproteinase (MMP) activity in the plaque causes collagen degradation. As a result of all these, it is a known fact that a plaque that is poor in collagen and has a thin fibrin sheath with an increased content of activated leukocytes forms a sensitive (vulnerable) plaque [15]. With the latest technological developments and the use of intravascular ultrasonography in medicine, studies have been conducted to examine the atherosclerotic process in diabetics and, more importantly, to examine the characteristics of coronary plaques, aiming to detect and treat sensitive plaques without causing major cardiovascular problems.

In a study published by Stone et al. in 2011, the presence of diabetes mellitus requiring insulin or the detection of TCFA by IVUS caused a three-fold increase in the risk of developing major cardiovascular events within three years, independent from the percentage of angiographic stenosis and other clinical factors [16]. Again, in a study completed in 2011 in which 170 patients and 1096 coronary plaques were examined, it was determined that the presence of TCFA increased the probability of developing a major cardiovascular event eight times during an average follow-up period of 625 days [17].

The relationship between the presence of thin, fibrous encapsulated fibroatheroma and diabetes mellitus has also been the subject of many studies. In the study, which was published in 2000 and evaluated the responsible lesions of 310 patients with acute coronary syndrome with VH-IVUS, diabetes mellitus was observed to be the only independent risk factor for the presence of TCFA [18]. In a study in which 112 non-diabetic and 63 diabetic lesions were examined by VH-IVUS, the rate of fibroatheroma with thin fibrous caps and the area of necrotic nuclei in the minimal lumen area were found to be significantly higher in diabetic patients [19]. Similar to our findings, the percentage of fibrous fatty plaque cross-sectional area in the minimal lumen area was significantly higher in diabetics compared to non-diabetics. Another similar aspect was that the volume and percentage of fibrous adipose tissue were significantly higher in diabetic patients in the aforementioned study.

In another prospective study, 160 coronary lesions were examined with VH-IVUS, and the patients were followed for a mean follow-up of 30 months. During this period, 10 cases of acute coronary syndrome were seen, of which 10 lesions were considered responsible. When these lesions were examined retrospectively, it was seen that the percentage of fibrosis-fatty components was significantly higher in active plaques compared to stable plaques. It was concluded that the percentage of fibrosis-fatty components in plaques may be associated with vulnerability [20]. This finding supported and suggested that the increased FFA% detected in the MLA of diabetic patients in our study may have a high sensitivity in terms of developing acute coronary syndrome in this patient group.

The volume and percentage of fibrous fatty plaque increased in patients with metabolic syndrome, and it was concluded that this situation contributed to plaque vulnerability [21]. In patients with diabetes mellitus or metabolic syndrome, it was observed that the volume and percentage of fibrous fatty plaque were found to be significantly higher than in non-diabetic and non-insulin-resistant patients [22]. In our study, in addition to the FFA% in MLA, the percentage of fibrous fatty plaque volume in the entire examination area in the coronary artery in the diabetic group tended to be higher, but this difference did not reach statistical significance (p =0.060).

Diabetic plaques had a higher rate of necrotic nuclei than non-diabetics, and the frequency of TCFA was higher in diabetic patients [23]. In our study, although TCFA was found to be more common in the diabetic group and HbA1C level was associated with the NC/DC volume ratio, the rate of necrotic nuclei was not higher in diabetic patients. Missel et al. evaluated all coronary arteries of 225 acute coronary syndrome patients with IVUS in 2008. As a result of this evaluation, they determined that the ST depression detected in the ECG and blood creatine kinase-myocardial band (CK-MB) values were directly proportional to the NC/DC volume ratio detected in the IVUS [24]. Again, in another study by Missel et al. on 473 male patients in 2007, they reported that the only VH-IVUS finding correlated with known markers of sudden cardiac death was the NC/DC ratio [25]. The relationship between the HbA1C-NC/DC ratio in our study can also be considered a similar finding in this direction.

In the related study of Kato et al., in which coronary lesions were examined by optical coherence tomography (OCT) in diabetic patients, TCFA was found to be high in patients with HbA1C levels of eight and above [26]. In our study, although no relationship was found between HbA1C levels and the presence of TCFA, the relationship between the presence of attenuated plaque and the NC/DC ratio and HbA1C levels, which are other possible indicators of the vulnerability of coronary plaque, was found to be significant. In addition to this finding, the detection of HbA1C>7.5 indicated the presence of attenuated plaque with 65% sensitivity and 90% specificity. It is very likely that this relative difference in findings is due to the different definitions of TCFA with OCT and VH-IVUS.

When the diabetic group was evaluated among themselves, it was observed that the rate of TCFA was significantly increased in diabetic patients who did not use insulin. Insulin plays an important role in many aspects of endothelial function, such as nitric oxide production. Nitric oxide is the main factor mediating endothelium-dependent relaxation in arteries and inhibiting platelet aggregation, cell adhesion, and smooth muscle cell proliferation. The recommendation to switch to the use of insulin in coronary artery patients with diabetes mellitus (a macroangiopathic complication) is now a classic piece of knowledge in cardiology books. This finding of our study is actually a new finding that supports the accuracy of the recommendation to switch to insulin in cases of coronary artery disease in diabetic patients, even though there was not a supportive study in the literature. In the future, it will be useful to support this information with more extensive IVUS studies.

Our other examination parameters were related to grayscale classical IVUS parameters and newly defined plaque features. The presence of negative remodeling in diabetics has been detected in many studies to date.

Especially after the discovery of attenuated plaques in grayscale IVUS examinations, it attracted the attention of the cardiology community, and many studies have been carried out on this subject until today. In a study conducted on 36 patients with acute coronary syndrome, it was determined that the presence of attenuated plaque in IVUS was strongly associated with the post-percutaneous coronary intervention (PCI) no-reflow phenomenon [27]. Again, in the study of the same group in 2010 on 47 attenuated plaques, it was found that the volume of necrotic nuclei in attenuated plaque areas was quite high, and these lesions were a sign of the presence of TCFA when examined with VH-IVUS [28]. Therefore, the presence of attenuated plaque on the gray scale is also an indicator of plaque vulnerability. High HbA1C levels indicate the presence of uncontrolled diabetes mellitus, and this is known to increase cardiovascular mortality. Therefore, we think that the relationship between HbA1C levels and the presence of attenuated plaque is a very important finding in defining the vulnerable patient [29]. Our study was not the first IVUS study performed on diabetic patients; however, to the best of our knowledge, this was the first study showing the relationship between HbA1C levels and the presence of attenuated plaque in the literature [30].

## Conclusions

We can list the results we reached in our study, in which diabetic and non-diabetic coronary plaques were compared in terms of basic vulnerability criteria. The incidence of thin fibrous encapsulated fibroatheroma (an important intravascular tomographic vulnerability criterion) was significantly higher in the diabetic patient group compared to non-diabetic patients. The FFA% measured in the narrowest lumen area determined by IVUS was significantly higher in diabetic patients than in non-diabetic patients.

As a result of the evaluation of coronary artery lesions with virtual ultrasonographic criteria, it was determined that plaque sensitivity was increased in diabetic patients (especially in diabetics with uncontrolled HbA1C levels and not using insulin). Considering the parallelism of increased plaque sensitivity with increased patient sensitivity, it can be thought that diabetes mellitus causes cardiovascular vulnerabilities by changing the morphology of coronary plaques.
